# Pre‐ and post‐conditioning with poly I:C exerts neuroprotective effect against cerebral ischemia injury in animal models: A systematic review and meta‐analysis

**DOI:** 10.1111/cns.13851

**Published:** 2022-05-05

**Authors:** Zeeshan Ahmad Khan, Dewan Md. Sumsuzzman, Jeonghyun Choi, George Kamenos, Yonggeun Hong

**Affiliations:** ^1^ Department of Physical Therapy College of Healthcare Medical Science & Engineering Gimhae Korea; ^2^ 26719 Biohealth Products Research Center (BPRC) Inje University Gimhae Korea; ^3^ 26719 Research Center for Aged‐life Redesign (RCAR) Inje University Gimhae Korea; ^4^ Department of Rehabilitation Science Graduate School of Inje University Gimhae Korea

**Keywords:** cerebral ischemia, meta‐analysis, poly I:C, systematic review, toll‐like receptors

## Abstract

**Background:**

Toll‐like receptor (TLR) agonist polyinosinic–polycytidylic acid (poly I:C) exerts neuroprotective effects against cerebral ischemia (CI), but concrete evidence supporting its exact mechanism of action is unclear.

**Methods:**

We evaluated the neuroprotective role of poly I:C by assessing CI indicators such as brain infarct volume (BIV), neurological deficit score (N.S.), and signaling pathway proteins. Moreover, we performed a narrative review to illustrate the mechanism of action of TLRs and their role in CI. Our search identified 164 articles and 10 met the inclusion criterion.

**Results:**

Poly I:C reduces BIV and N.S. (*p* = 0.00 and *p* = 0.03). Interestingly, both pre‐ and post‐conditioning decrease BIV (preC *p* = 0.04 and postC *p* = 0.00) and N.S. (preC *p* = 0.03 and postC *p* = 0.00). Furthermore, poly I:C upregulates TLR3 [SMD = 0.64; CIs (0.56, 0.72); *p* = 0.00], downregulates nuclear factor‐κB (NF‐κB) [SMD = −1.78; CIs (−2.67, −0.88); *p* = 0.0)], and tumor necrosis factor alpha (TNF‐α) [SMD = −16.83; CIs (−22.63, −11.02); *p* = 0.00].

**Conclusion:**

We showed that poly I:C is neuroprotective and acts via the TLR3/NF‐κB/TNF‐α pathway. Our review indicated that suppressing TLR 2/4 may illicit neuroprotection against CI. Further research on simultaneous activation of TLR3 with poly I:C and suppression of TLR 2/4 might open new vistas for the development of therapeutics against CI.

## Graphical abstract

Polyinosinic–polycytidylic acid (poly I:C) has shown promise as a neuroprotective agent in rodents with induced ischemic stroke in both adults and neonates. Previous studies have focused on either preconditioning (preC) or post‐conditioning (postC) leading to ambiguity in determining the neuroprotective potential of poly I:C. This study showed a combined as well as a separate meta‐analysis of preC and postC of poly I:C against cerebral ischemia (CI). Interestingly, we observed that both preC and postC offer neuroprotection against CI and act via the TLR3/NF‐κB/TNF‐α pathway.

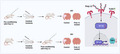

## 1 INTRODUCTION

Cerebral ischemia (CI) is a condition where blockage in an artery limits the distribution of oxygen‐rich blood to the brain, and this leads to brain tissue damage. Cerebral ischemia may be caused by cardiac arrest,^1^ stroke,^2^ severe anemia,^3^ aging,^4^, ^5^ systemic hypotension and hypoxia,^6^, ^7^, ^8^ metabolic derangements,^9^, ^10^ or strangulation.^11^, ^12^ Severe CI such as stroke has a high death rate with approximately 795,000 people having a stroke each year,^13^ and survivors often showing significant neurological disabilities and cognitive decline.^14^, ^15^ Patients that survive severe CI bear an exorbitant financial burden, where total medical care cost associated with stroke for Americans will soon be around $3 trillion from 2005 to 2050 (inflation‐adjusted to 2021).^16^ If the high mortality and financial impact of CI are to be mitigated, novel therapy development targeted against CI is necessary.

Toll‐like receptors (TLRs) are expressed in astrocytes, microglia, and endothelial cells and are upregulated in the brain following ischemia.^17^ Although activation of TLRs signaling worsens stroke injury, some reports suggest that the TLR3 activation by polyinosinic‐polycytidylic acid (poly I:C) mediates innate immune responses and thereby provides neuroprotection.^18^, ^19^ On the other hand, no significant differences in indicators of neuroprotection such as brain infarct volume (BIV) and neurological deficit score (N.S.) between TLR3 knockout (KO) and wild‐type (WT) mice after CI was observed,^20^ indicating that the TLR3 signaling pathway might not be directly linked to ischemic brain injury.^21^ Thus, identifying the receptor and transduction pathways involved with TLR signaling is critical for outlining drug delivery methods.

A safe and effective form of the neuroprotective drug against CI remains elusive. Poly I:C, a synthetic analog of double‐stranded RNA (dsRNA), has shown promise as a neuroprotective agent in rodents with induced ischemic stroke in both adults^19^, ^22^, ^23^ and neonates.^24^, ^25^ However, Stridh et al.^26^ demonstrated that preconditioning (preC) of poly I:C might increase brain BIV and augment the loss of myelin basic protein by a factor of 5, which indicates an increased vulnerability of the neonatal brain to CI compared to control. Current literature on this topic is divided and the neuroprotective effect of poly I:C is currently inconclusive. Therefore, a meta‐analysis is warranted to estimate the therapeutic potential of poly I:C against CI.

Primarily, we set out to clarify the therapeutic potential and signal transduction pathways of poly I:C following CI in rodent models. Brain infarct volume, N.S., cell death, and level of poly I:C‐associated pathway protein were chosen as outcomes for our meta‐analysis to measure the therapeutic potential of poly I:C. Secondly, as other TLRs can be a potential therapeutic target against CI, we narratively reviewed various signaling pathway of TLR and their role in stroke.

## 2 METHODS

### 2.1 Search strategy and inclusion criteria

Research articles reporting on the intervention of poly I:C in ischemic animal models were included in this systematic review and meta‐analysis. The literature search was executed using keywords such as “poly I:C” in combination with “ischemia, reperfusion, hypoxia,” in PubMed, and Embase, for studies published up until March 8, 2021. References (reverse citation tracking) and citations (forward citation tracking) of included studies were examined to identify any remaining studies or unpublished studies such as preprints that might be missing from our literature search results. The search strategies are given in Table S1 and S2. No limits on language or publication date were used. The full inclusion criteria used in our study are available in Appendix 1.

### 2.2 Study selection and data extraction

Two authors (Z.A.K. and D.M.S.) individually screened relevant articles by title and abstract, and full texts to identify articles that fulfilled our eligibility criteria. Disagreements regarding study selection were settled by a discussion with a third and fourth author (J.C. and G.C.K.).

Two authors (Z.A.K. and D.M.S.) individually extracted the data from selected studies. Information related to the authors, country, year of publication, sex, age, sample size, and outcome measures was extracted. The full details of data extraction used in our study are available in Appendix 1.

### 2.3 Quality assessment of the selected article

To assess various aspects of bias related to animal intervention‐based studies, the risk of bias (RoB) was estimated by two reviewers independently using the SYRCLE RoB tool (Cochrane RoB tool).^27^, ^28^ The RoB tool covers 10 items related to six different types of bias including selection, performance, detection, attrition, reporting, and other bias. Responses of “yes,” “no,” and “unsure” indicated low, high, and unclear RoB, respectively.

### 2.4 Analysis of extracted data

The extracted data from the selected studies were then entered into Stata SE 16 software. For effect size analysis, the standardized mean difference (SMD; Hedges'g) was used when studies assessed the same outcome by different measurements.^29^, ^30^ The mean difference (MD) was used whenever the outcome measures of associated studies utilized the same scale without other significant differences.^31^ The detailed data analysis methods used in our study are available in Appendix 1.

## 3 RESULTS

### 3.1 Study search and selection

**FIGURE 1:**
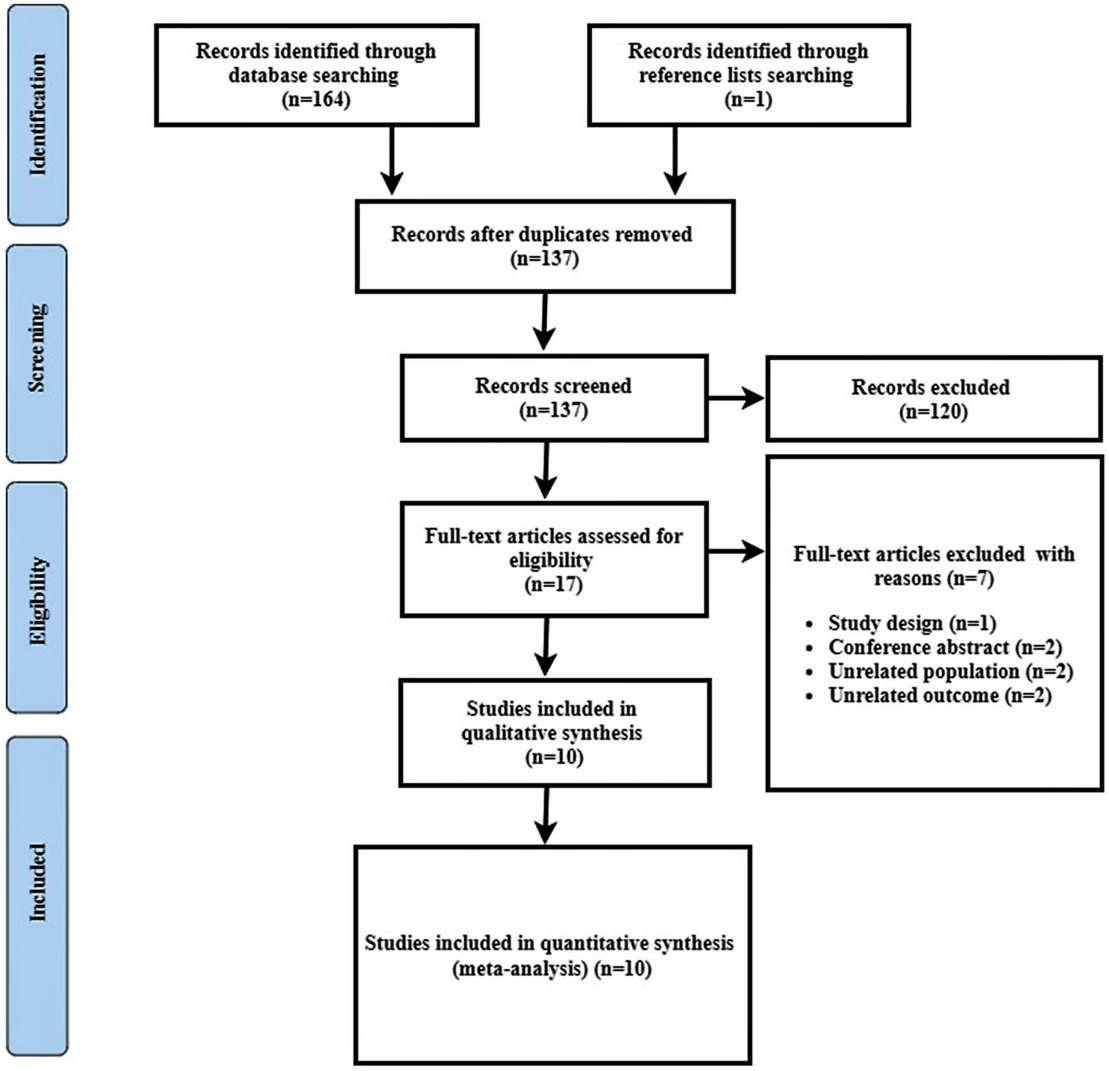
Flow diagram of study search and retrieval process

In total, 164 studies were found through our PubMed and Embase electronic database search. Duplicate studies were removed, 137 potentially relevant articles were screened based on title and abstract screening, and 120 articles were excluded. The full‐text screening was performed with the remaining 17 studies and 7 studies were excluded due to: unrelated outcomes (*n* =  2), conference abstract (*n* =  2) inappropriate study design (*n* = 1), and unrelated population (*n* =  2) (Table S3). Finally, 10 studies^19^, ^21^, ^22^, ^23^, ^24^, ^25^, ^26^, ^32^, ^33^, ^34^ fulfilled our eligibility criteria and were selected for systematic review and meta‐analysis (Figure 1).

### 3.2 Study characteristics and risk of bias assessment

**FIGURE 2:**
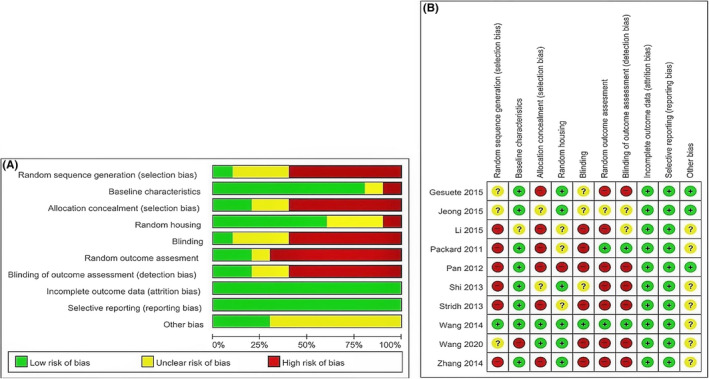
Risk of bias. (A) Overall RoB for each item in the SYRCLE tool for all included studies. Each RoB item is presented as a percentage based on all included studies. (B) Individual RoB for each of the included animal studies. Each item in the SYRCLE tool was scored as “yes,” “no,” or “unclear”

The key characteristics of the studies included for systematic review and meta‐analysis are presented in Table 1. The included studies were published between 2011 and 2020. MCAo was used to induce brain ischemia in 8 of the 10 included studies,^19^, ^21^, ^22^, ^23^, ^25^, ^32^, ^33^, ^34^ while two studies used carotid artery ligation.^24^, ^26^ All the included studies used male animals except Stridh et al.,^26^ who used both male and female mice. Eight studies used mice,^19^, ^21^, ^24^, ^25^, ^26^, ^32^, ^33^, ^34^ only one study used rats^23^ and both mice and rats were used in one study.^22^ The four studies were from the USA^21^, ^25^, ^32^, ^33^ and China,^19^, ^22^, ^23^, ^34^ while one study was from Canada^24^ and Sweden,^26^ respectively. Eight studies used an intraperitoneal injection of poly I:C,^21^, ^22^, ^23^, ^24^, ^26^, ^32^, ^33^, ^34^ one used intramuscular^19^ and subcutaneous^25^ each. Various concentrations of the poly I:C ranging from 0.3 to 4 mg/kg were used in the included studies. The RoB analysis summary and individual RoB scores have shown in Figure 2A,B, and a detailed description of RoB is available in Appendix 1.

**TABLE 1:** Characteristics of the included studies

Author (Year)	Country	Sex	Species	Poly I:C Dose (mg/kg)	Intervention time	Delivery route	CI method	Evaluated parameters
Packard^25^	USA	male	Mice	1.6	72 h before CI	Subcutaneous	MCAo	Brain infarct volume (TTC staining), and N.S. (corner test)
Pan^19^	China	N/A	Mice	0.3	2 h before CI	Intramuscular	MCAo	Brain infarct volume (TTC staining), N.S. (Zea‐Longa), and TNFα (ELISA)
Shi^24^	Canada	N/A	Rat	0.3	48 h before CI	I.P.	CAo	Brain infarct volume (H‐E staining), IRF3 and NF‐κB protein level (Western blot)
Stridh^26^	Sweden	Male/Female	Mice	5	14 h before CI	I.P.	CAo	Brain infarct volume (MAP−2‐negative area in the ipsilateral hemisphere)
Wang^22^	China	Male	Mice/rat	1.25	3 h after CI	I.P.	MCAo	Brain infarct volume (TTC staining), N.S. (spontaneous activity, symmetry of movement, open‐field path linearity, vibrissae touch; postural reflex test), cell death (TUNEL assay), TNFα (ELISA), IFR3, NF‐κB, and TLR3 protein level (Western blot)
Zhang^32^	USA	Male	Mice	4	1 h before CI	I.P.	MCAo	Brain infarct volume (TTC staining), Cell death (TUNEL assay), and IFR3, NF‐κB protein level (Western blot)
Gesuete^21^	USA	male	Mice	3	24 h before CI	I.P.	MCAo	Brain infarct volume (TTC staining)
Jeong^33^	USA	male	Mice	1	After CI	I.P.	MCAo	Brain infarct volume (TTC staining), TLR−3 protein level (Western blot)
Li^23^	China	male	Rat	1.25	1, 3, and 5 days after CI	I.P.	MCAo	Brain infarct volume (TTC staining), GFAP protein level (Western blot)
Wang^34^	China	male	Mice	1.25	24 h before CI	I.P.	MCAo	Brain infarct volume (TTC staining), N.S. (hangs onto string with forepaws, hindpaw(s) and tail; hangs onto string with forepaws and hindpaw(s); hangs on with forepaws and moves laterally on string; hangs on with forepaw(s); falls off within 2 s), Cell death (TUNEL assay), and GFAP protein level (immunostaining).

Abbreviations: CI, Cerebral ischemia; CAo, carotid artery occlusion; H‐E staining, hematoxylin and eosin staining; MCAo, middle cerebral artery occlusion; MAP‐2, microtubule‐associated protein‐2; I.P., intraperitoneal; poly I:C, Polyinosinic–polycytidylic acid, TNFα, tumor necrosis factor alpha; TLR3, toll‐like receptor 3; GFAP, glial fibrillary acidic protein; IFR3, interferon regulatory factor 3; NF‐κB, nuclear factor‐κB; TTC staining, 2,3,5‐triphenyltetrazolium chloride staining; UK, United Kingdom; USA, United States of America.

### 3.3 Meta‐analysis

#### 3.3.1 Effect of poly I:C intervention on brain infarct volume, neurological scores, and brain cell death

**FIGURE 3:**
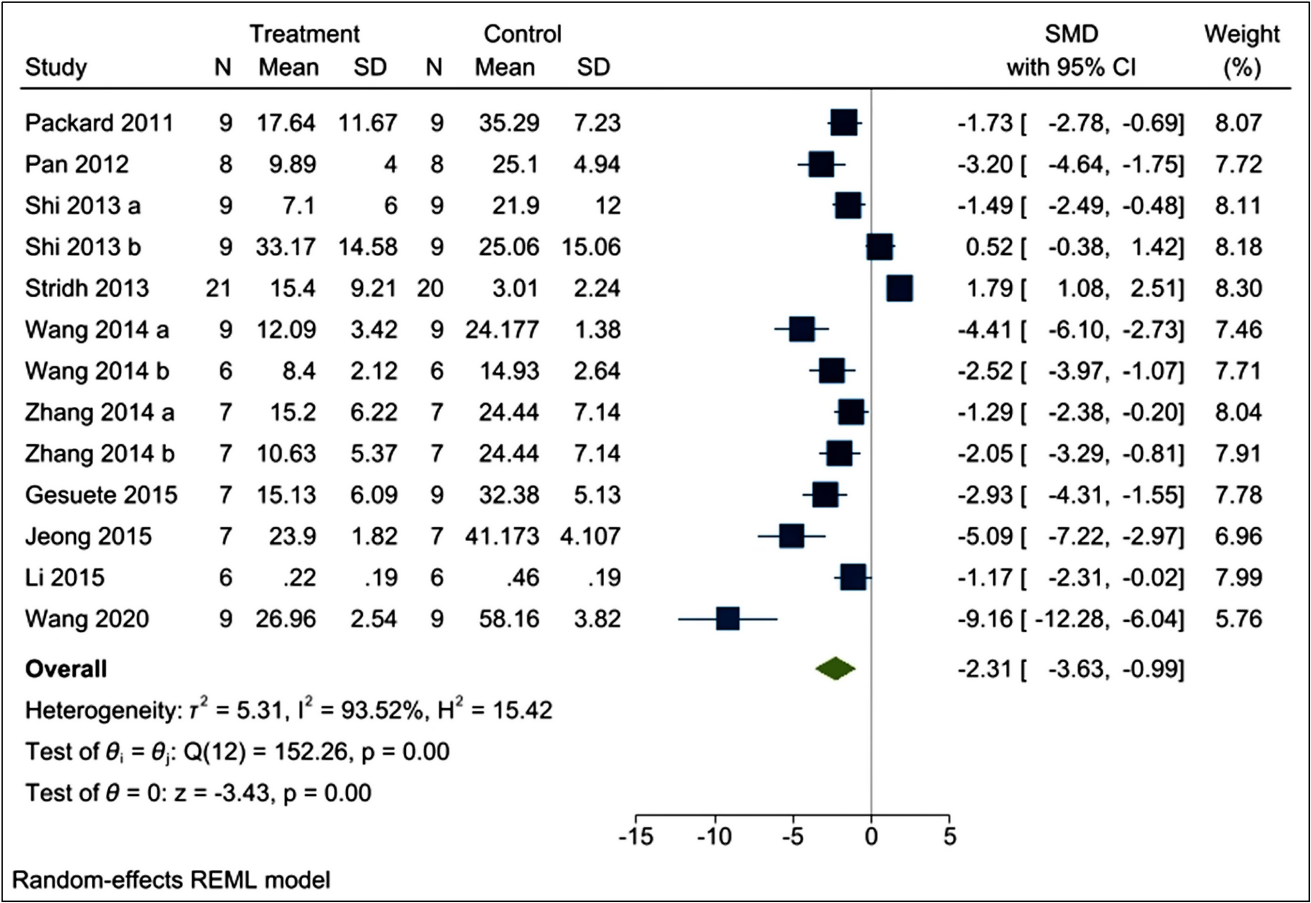
Forest plot comparing changes in BIV between poly I:C and vehicle‐treated groups. Compared with vehicle treatment, BIV was significantly reduced in the poly I:C group. The prism represents the overall statistical results of the experimental data, squares represent the weight of each study, and horizontal lines represent the 95% CIs for each study. Normality of BIV was checked using the Shapiro–Wilk test (*p* = 0.76329). BIV, brain infarct volume; poly I:C, Polyinosinic:polycytidylic acid; CIs, Confidence intervals; SD, Standard deviation; IV, Independent variable

**FIGURE 4:**
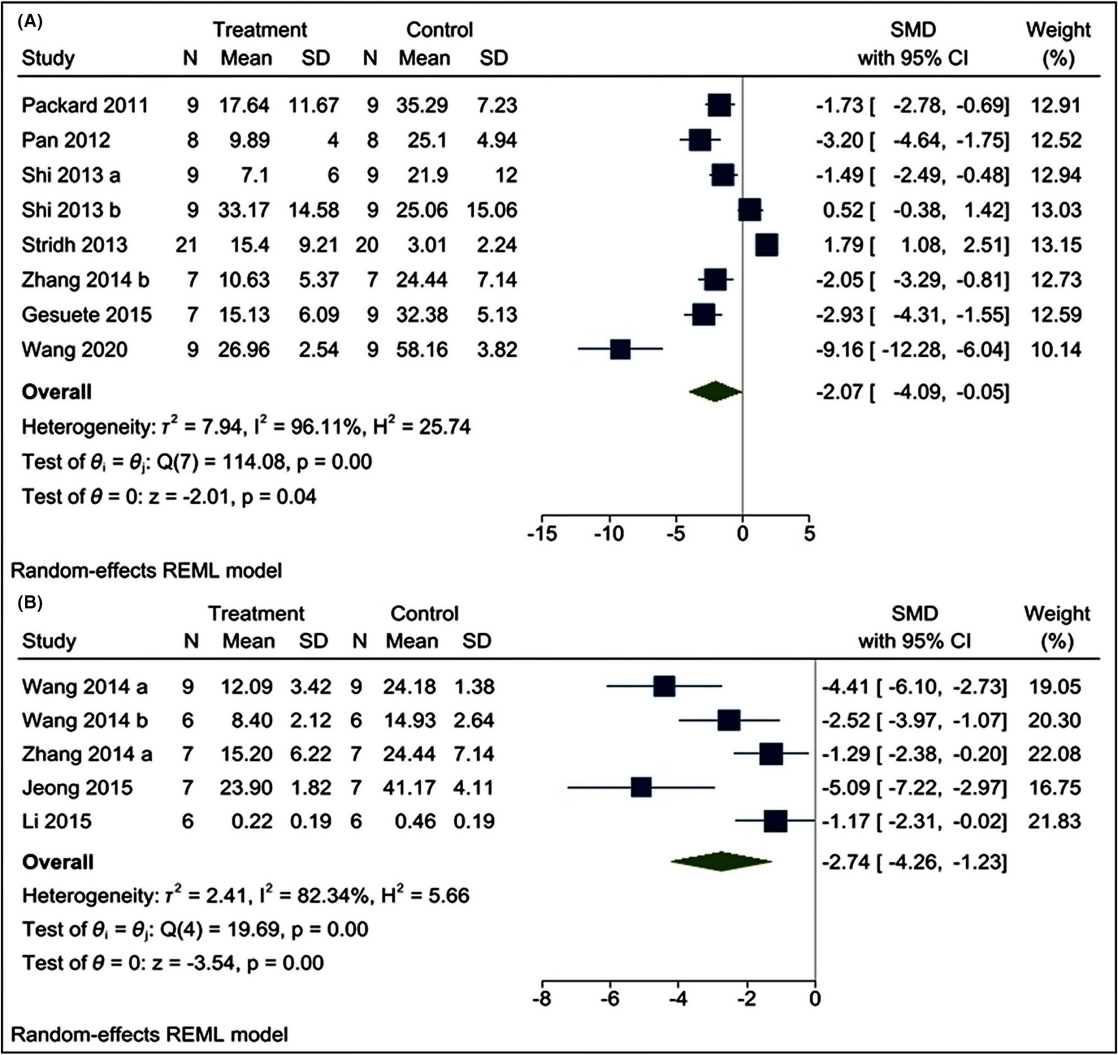
Forest plot comparing changes in BIV between (A) preconditioning and (B) Post‐conditioning with poly I:C and vehicle‐treated groups. Compared with vehicle treatment, both pre‐ and post‐conditioning significantly reduced the BIV. The prism represents the overall statistical results of the experimental data, squares represent the weight of each study, and horizontal lines represent the 95% CIs for each study. BIV, brain infarct volume; poly I:C, Polyinosinic:polycytidylic acid; CIs, Confidence intervals; SD, Standard deviation; IV, Independent variable

The effects of poly I:C intervention on BIV after CI were determined in all the included research articles.^19^, ^21^, ^22^, ^23^, ^24^, ^25^, ^26^, ^32^, ^33^, ^34^ In all the included studies, BIV was estimated by 2,3,5‐triphenyl tetrazolium chloride (TTC) staining^19^, ^21^, ^22^, ^23^, ^25^, ^32^, ^33^, ^34^ except Shi et al.^24^ and Stridh et al.^26^ who used hematoxylin and eosin (H‐E) staining and immunohistochemistry of microtubule‐associated protein‐2 (MAP‐2), respectively. Using a random‐effects model, we found that poly I:C significantly reduced BIV levels [I^2^ = 93.52%; SMD = –2.31; CIs (−3.63, −0.99); *p* = 0.00] (Figure 3). Moreover, we find that both preC [I^2^ = 96.11%; SMD = −2.07; CIs −4.09, −0.05); *p* = 0.04] and post‐conditioning (postC) [I^2^ = 82.34%; SMD = −2.74; CIs (−4.26, −1.230); *p* = 0.00] with poly I:C is neuroprotective against CI (Figure 4). We also performed meta‐regression of the BIV with various doses of poly I:C and found no correlation between them (*R* ^2^ = 0.54 and *p* = 0.19) (Figure S1).

**FIGURE 5:**
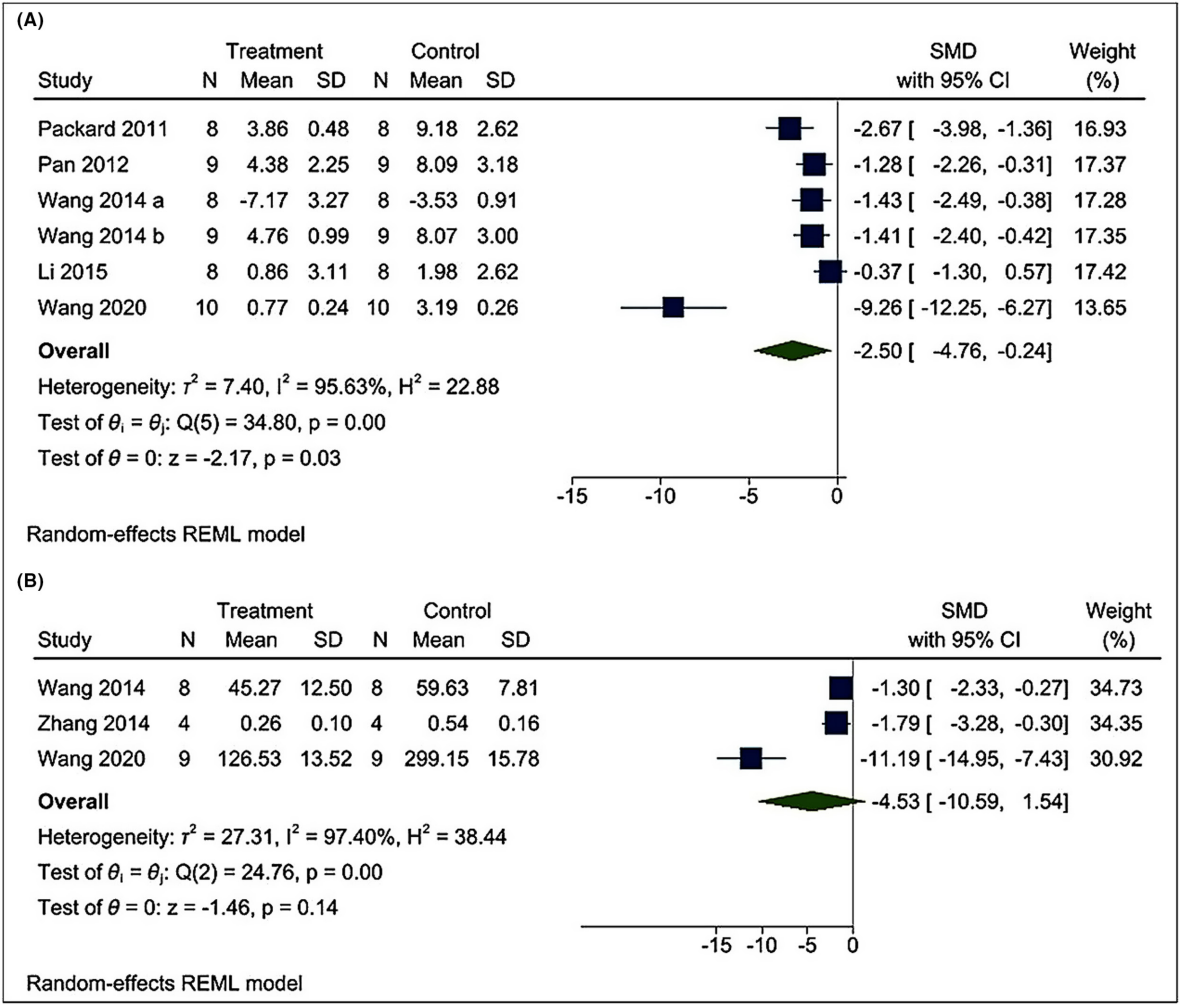
Forest plot comparing changes in N.S. and cell death between poly I:C and vehicle‐treated groups following cerebral ischemia. Compared with vehicle treatment, (A) N.S. was significantly reduced, while no effect on (B) cell death was observed in the poly I:C group. The prism represents the overall statistical results of the experimental data, squares represent the weight of each study, and horizontal lines represent the 95% CIs for each study. Normality of N.S. (*p* = 0.05777) and cell death (*p* = 0.68631) were checked using the Shapiro–Wilk test. N.S., Neurological deficit score; Poly I:C, Polyinosinic:polycytidylic acid; CIs, Confidence intervals; SD, Standard deviation; IV, Independent variable

**FIGURE 6:**
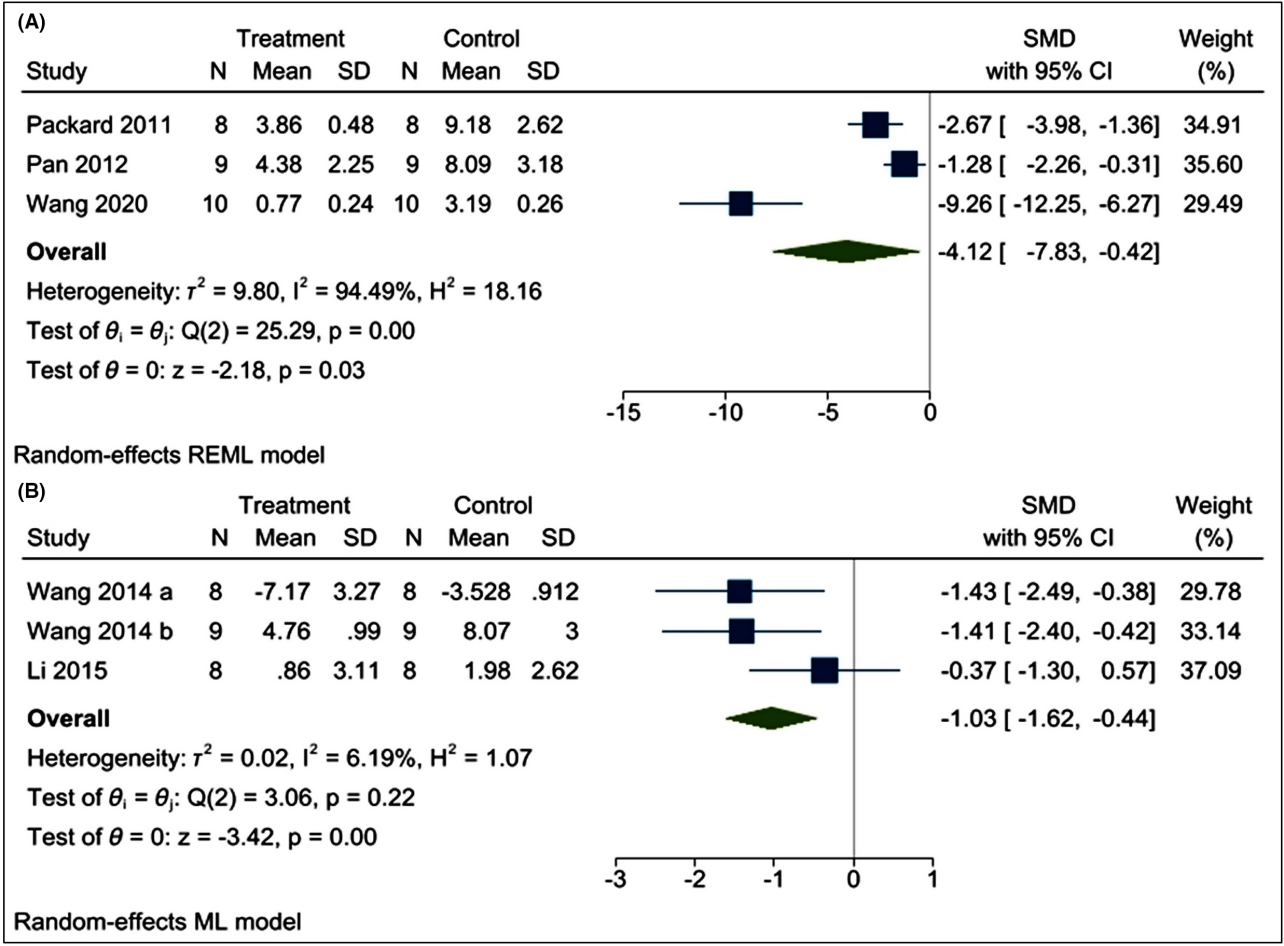
Forest plot comparing changes in N.S. between (A) preconditioning and (B) post‐ conditioning with poly I:C and vehicle‐treated groups. Compared with vehicle treatment, BIV was significantly reduced in both poly I:C groups. The prism represents the overall statistical results of the experimental data, squares represent the weight of each study, and horizontal lines represent the 95% CIs for each study. N.S., neurological deficit score; Poly I:C, polyinosinic:polycytidylic acid; CIs, confidence intervals; SD, standard deviation; IV, independent variable

The positive correlation between BIV and the N.S. is well established.^35^ Our N.S. meta‐analysis with poly I:C and CI consist of five studies.^19^, ^22^, ^23^, ^25^, ^34^ Using a random‐effects model, we found that poly I:C reduces the N.S., which is an indicator of better functional recovery [I^2^ = 95.63%; SMD = –2.50; CIs (−4.76, −0.24); *p* = 0.03] (Figure 5A). The effects of poly I:C on cell death were assessed from three studies.^22^, ^34^ The cell death was measured by TUNEL assay. Using a random‐effects model, we found that poly I:C intervention did not affect cell death [I^2^ = 97.40%; SMD = −4.53; CI (−10.59, 1.54); *p* = 0.14] (Figure 5B). Furthermore, we found that both preC [I^2^ = 94.49%; SMD = −4.12; CIs (−7.83, −0.42); *p* = 0.03] and postC [I^2^ = 6.19%; SMD = −1.03; CIs (−1.62, −0.44); *p* = 0.00] with poly I:C lowers N.S. (Figure 6). Owing to high heterogeneity in both BIV and N.S. we performed a detailed subgroup and leave‐one publication/year out sensitivity analysis (Appendix 1). We also evaluated publication bias by visually examining the asymmetry in the funnel plot and Egger's test of asymmetry (Appendix 1).

#### 3.3.2 Effect of poly I:C on the level of TLR3, NF‐κB, and TNF‐α

**FIGURE 7:**
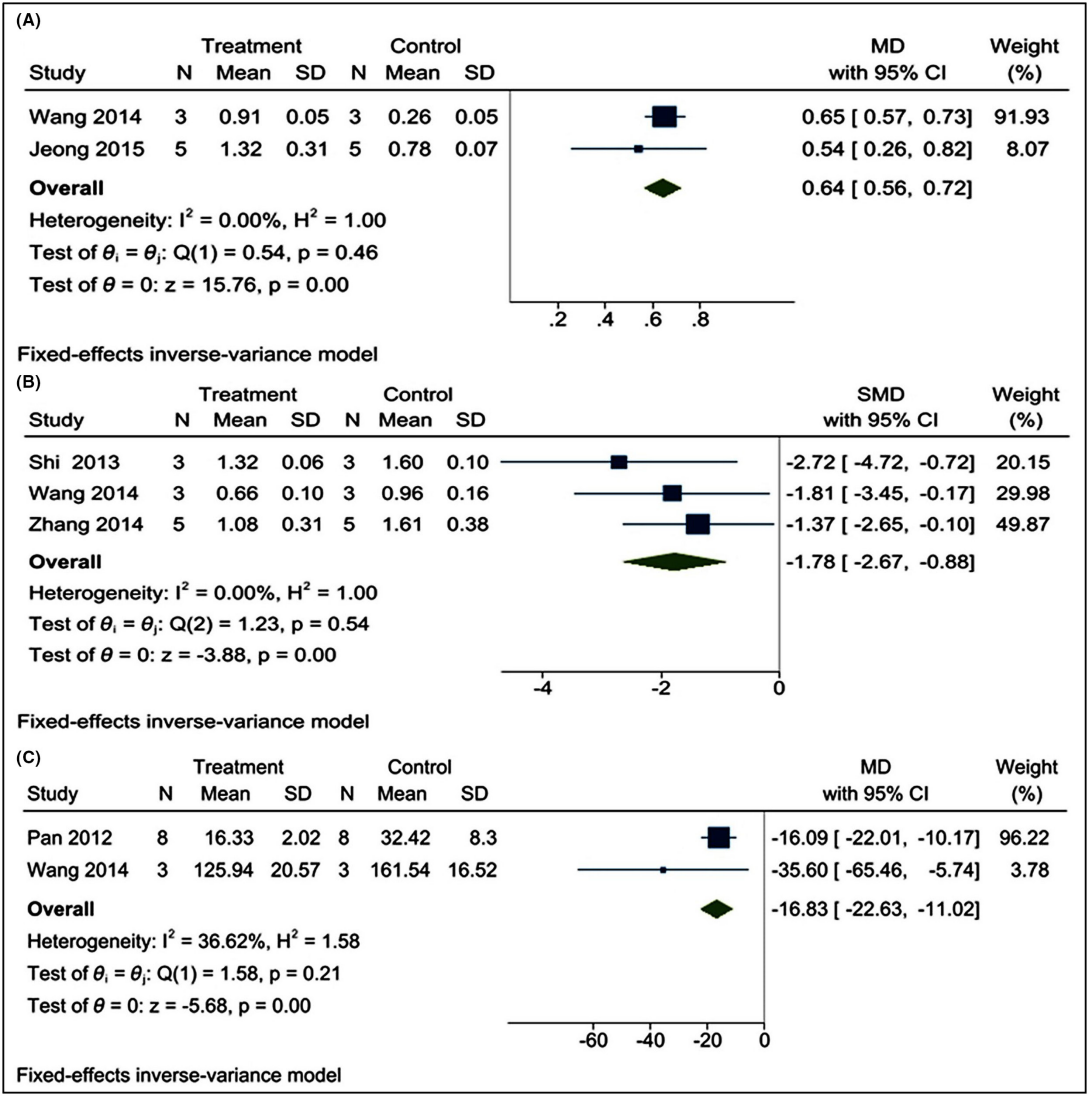
Forest plot comparing changes in TLR3, NF‐κB, and TNF‐α in the brain between poly I:C and vehicle‐treated groups following cerebral ischemia. Compared with vehicle treatment, (A) TLR3 was increased, (B) NF‐κB and (C) TNF‐α, were significantly reduced in the poly I:C group. The prism represents the overall statistical results of the experimental data, squares represent the weight of each study, and horizontal lines represent the 95% CIs for each study. Normality of NF‐κB was checked using the Shapiro–Wilk test (*p* = 0.70173). Poly I:C, polyinosinic:polycytidylic acid; TLR3, Toll‐like receptor 3; NF‐κB, nuclear factor‐κB; TNF‐α, tumor necrosis factor alpha; CIs, confidence intervals; SD, standard deviation; IV, independent variable

The change in TLR3 and NF‐κB level in the brain after CI injury was determined based on two^22^, ^33^ and three studies,^22^, ^24^, ^32^ respectively. All the studies quantified TLR3 by Western blot,^22^, ^33^ while NF‐κB level was measured by the western blot^22^, ^24^ and electrophoretic mobility shift assay.^32^ Using a fixed‐effects inverse variance model, we found that poly I:C level significantly upregulates the level of TLR3 [I^2^ = 0.00%; SMD = 0.64; CIs (0.56, 0.72); *p* = 0.00]. On the other hand, the level of NF‐κB protein was downregulated in the rodent brain after poly I:C treatment [I^2^ = 0.00%; SMD = −1.78; CIs (−2.67, −0.88); *p* = 0.00] (Figure 7A,B). Fixed‐effects inverse variance model analysis showed that poly I:C intervention significantly downregulates the level of TNF‐α in rodent brain [I^2^ = 36.62%; SMD = −16.83; CIs (−22.63, −11.02); *p* = 0.00] (Figure 7C).

#### 3.3.3 Effect of poly I:C on the interferon regulatory factor3 (IRF) and glial fibrillary acidic protein (GFAP)

**FIGURE 8:**
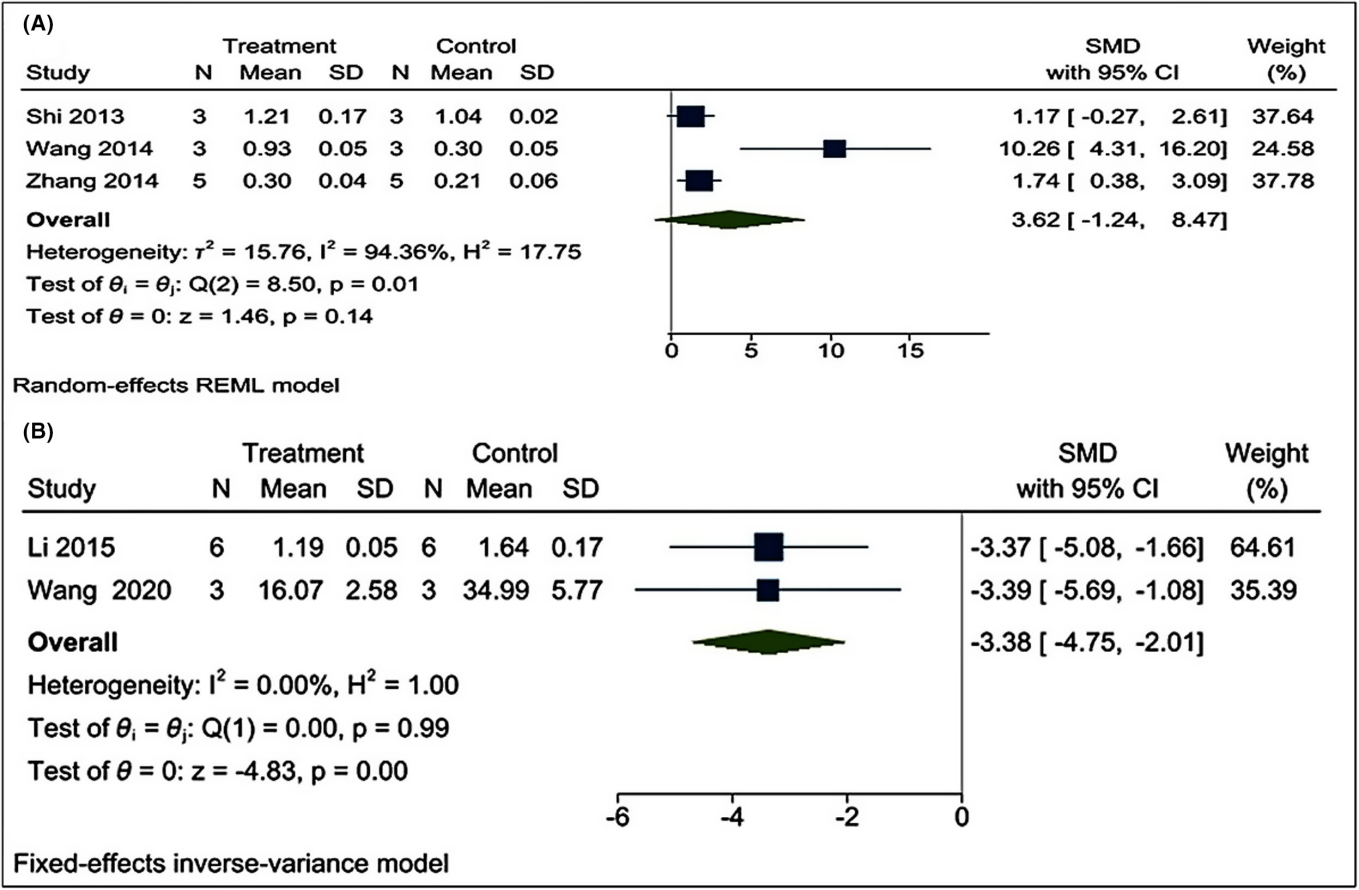
Forest plot comparing changes in IRF3 and GFAP protein level in brain between poly I:C and vehicle‐treated groups following cerebral ischemia. No change was observed in the (A) IRF3 protein level, while (B) GFAP protein was downregulated in the poly I:C group. The prism represents the overall statistical results of the experimental data, squares represent the weight of each study, and horizontal lines represent the 95% CIs for each study. Normality of IRF was checked using the Shapiro‐Wilk test (*p* = 0.57957). Poly I:C, polyinosinic:polycytidylic acid; IRF3, interferon regulatory factor 3; GFAP, glial fibrillary acidic protein; CIs, confidence intervals, CI; SD, standard deviation; IV, independent variable

Three^22^, ^24^, ^32^ and two^22^, ^23^ studies measured the effect of poly I:C treatment on the level of IRF3 and GFAP, respectively. Using a random‐effect model, we found that poly I:C has no effect on the IRF3 level in the brain after CI [I^2^ = 94.36%; SMD = 3.62; CIs (−1.24, 8.47); *p* = 0.14] (Figure 8A). The reason behind the high heterogeneity in IRF3 results was explored by leaving one publication/year out (Appendix 1). On the other hand, we applied fixed‐effects inverse variance model and found that poly I:C significantly downregulated the level of GFAP in the brain after CI [I^2^ = 0.00%; SMD = −3.38; CIs (−4.75, −2.01); *p* = 0.00] (Figure 8B).

### 3.4 A systematic review of effect poly I:C intervention on apoptosis‐related proteins after CI

Neuronal apoptosis plays a key role in CI injury.^36^ Owing to the anti‐apoptotic effect Bcl‐2 is vital for cell survival, whereas Bax promotes apoptosis. Wang et al.^22^ demonstrated that poly I:C treatment following CI significantly upregulates the Bcl‐2 and downregulates the level of Bax. Enhanced caspase‐3 and caspase‐8 activities are other key markers of apoptosis.^37^ Zhang et al.^32^ showed that poly I:C prevented hypoxia ischemia‐induced caspase‐3 and −8 activity in microglial cells, indicating that poly I:C may attenuate microglial activation and apoptosis in response to ischemic stimulation. The results further support the hypothesis that the neuroprotective effect of poly I:C treatment is due to the reduction of apoptosis.

Cell death or survival pathways were conjointly affected with a rise in expression of the apoptosis‐associated factor Fas, whereas pro‐survival pathways including AKT phosphorylation were reduced. As opposed to other neuroprotective reports, Stridh et al.^26^ showed that poly I:C treatment transiently downregulated the Akt phosphorylation and upregulated Fas ligand mRNA in the neonatal rodent ischemia model. This finding was in agreement with the study showing that poly I:C exacerbated neurodegeneration by the upregulation of Fas ligand.^38^ These results indicate that poly I:C might negatively impact cell survival.

### 3.5 A narrative review of TLR signaling pathways

**FIGURE 9:**
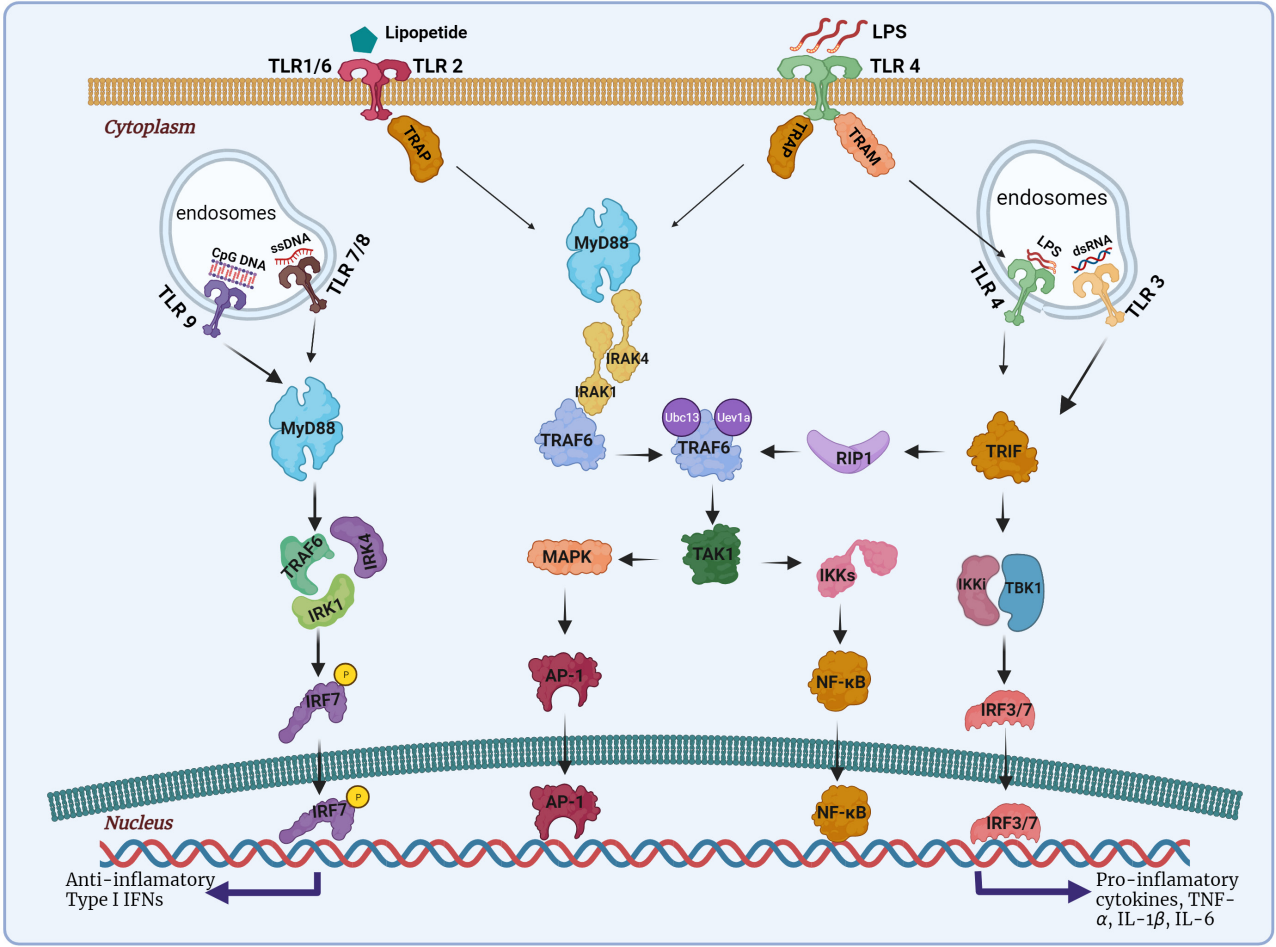
Toll‐like receptor cell signaling pathways. Every TLR family member, except TLR3, uses the MyD88‐dependent signaling pathway. TRAP bridges MyD88 to TLR1/6/2/4 and felicitates their interactions with TRAF6. TRAF6 activates the complex of TAK1 which activates both the IKK complex and MAPK. IKKs induce translocation of NF‐κB to the nucleus where pro‐inflammatory gene expression is triggered. MAPKs cause the AP‐1 nuclear translocation. TLR 7/8 and 9 activate MyD88 initiates the translocation of IRF7 to the nucleus and leads to IFN expression. TLR3 and TLR4 recruit TRIF and lead to translocation of IRF3/IRF7 and NF‐κB to the nucleus resulting in the expression of various IFNs and cytokines. Furthermore, TRIF can interact with TRAF6 through an effect on RIP‐1, which is followed by NF‐κB activation and production of the proinflammatory complex. TLR, Toll‐like receptor; MyD88, myeloid differentiation primary response gene 88; TRAP, transmembrane adaptor protein; TAK1, transforming growth factor beta‐activated kinase 1; MAPK, mitogen‐activated protein kinases; IKKs, inhibitory kappa B kinases; NF‐κB, nuclear factor‐kappa B; AP‐1, activator protein 1; IRF7, interferon regulatory factor 3; IFNs, interferons; RIP‐1, receptor‐interacting serine/threonine–protein kinase 1

Eleven types of TLRs are known to exist in humans, with 13 in animals.^39^ Initial TLR signaling begins when corresponding ligands engage with their respective receptors, and signaling concludes with the production of cytokines and chemokines. Toll‐like receptors have five different adaptor molecules: MyD88, MyD88 adaptor‐like protein (MAL), TIR‐domain‐containing adapter‐inducing interferon‐β (TRIF), TRIF‐related adaptor molecule (TRAM), and sterile‐alpha and armadillo motif‐containing protein (SARM).^40^ Adaptor molecules recruit downstream kinases and transcription factors involved in inflammatory and antiviral responses. Toll‐like receptors can converge on one of the two‐second messenger pathways based on the recruitment of specific adaptors: MyD88‐dependent or MyD88‐independent (Figure 9).^41^

Bridging of MyD88 to TLR1/2/4/6 transpires through TRAP/Mal adaptor proteins and facilitates their interaction with TRAF6. Following TIRAP/Mal assembly, TLR1/2/4/6 is linked to MyD88, and MyD88 can subsequently activate the interleukin (IL)‐1 receptor‐associated kinase 4 (IRAK4). Subsequently, MyD88 activates the IL‐1 receptor‐associated kinase 1 and 4 (IRAK 1 and 4).^41^, ^42^ The MyD88 and IRAKs complexes dissociate and interact with tumor necrosis factor receptor‐associated factor 6 (TRAF6). Afterward, the TRAF6 is ubiquitinated by ubiquitin‐conjugating enzyme 13 (Ubc13) and ubiquitin‐conjugating enzyme E2 variant 1 (Uev1A), which triggers the activation of a complex containing transforming growth factor beta‐activated kinase 1 (TAK1).^41^, ^42^ This TAK1 activates inhibitory kappa B kinases (IκKs) including IκK‐α, IκK‐β, and IκK‐γ like proteins. This complex induces nuclear translocation of dissociated NF‐κB and subsequent production of pro‐inflammatory cytokine genes; TNF‐α, IL‐1, and IL‐6. Simultaneous to IκK activation, TAK1 activates mitogen‐activated protein kinases (MAPKs), which are comprised of three p38 MAPK groups, extracellular signal‐regulated kinases (ERKs), and c‐Jun‐N‐terminal kinases.^41^, ^42^ The MAPK is responsible for the expression of activator protein 1 (AP‐1), a transcription factor complex that regulates the production of cytokine and growth factors in response to a variety of stimuli; including stress, bacterial, and viral infections (Figure 9).^43^

Toll‐like receptor 4 is the sole member of the TLR family capable of activating genes involved in the MyD88 and TRIF signaling pathways.^44^ Lipopolysaccharide (LPS) is a ligand that binds to TLR4, causes endosome formation, culminating in TRAM translocation to the cytosol and activation of the TRIF‐dependent signaling cascade, with the ability of TRIF to be activated by TLR3 as well. Subsequently, TRIF induces activation of the TANK‐binding kinase/IB kinase (TBK1/IκKi) complex, resulting in phosphorylation of the IRF 3, and IRF7 in this pathway.^44^ Phosphorylated IRF3 and IRF7 dimers subsequently translocate to the nucleus and induce cytokine expression. These TRIF also activate receptor‐interacting protein 1, which aids in the ubiquitination of TRAF6 leading to activation of NF‐κB and AP‐1 expression.

Toll‐like receptors 7/8/9 reside in endosomal vesicles, which also act through MyD88.^45^, ^46^ Upon activation by ssRNA (TLR7 and 8) or CpG‐rich DNA (TLR9), the respective TLRs activate MyD88, which induces the formation of TRAF6, IRAK1, and IRAK4 complexes.^45^, ^46^, ^47^ Phosphorylation of IRF7 complexes leads to its nuclear translocation and production of IFNs (Figure 9).

#### 3.5.1 Toll‐like receptors in CI

Toll‐like receptors and their ligands play a significant role in the repercussion of brain ischemia. Toll‐like receptors mediate inflammatory responses in immune cells, suggesting that these receptors aid in causing ischemia damage. After brain ischemia, astrocytes and microglia recognize injury‐associated modulators and instigate inflammatory cascades. Following that, immune cells such as macrophages and neutrophils infiltrate the injured region, releasing a large number of inflammatory cytokines, proteolytic enzymes, and other cytotoxic mediators. Additionally, Rayasam et al.^48^ showed a crucial role of the TLR3‐neutrophil axis in disrupting the structure and function of vascular networks by neutrophil elastase (NE) activation and NE‐induced neutrophil extracellular trap formation. TLR3 appears to be implicated in brain ischemia. The totality of TLR3 signaling is widely explored in the meta‐analysis section; therefore, our focus will be on other TLRs which play a critical role in stroke.

The role of TLRs in CI is well studied and mostly intended to determine which TLR subpopulations are required for the development of ischemic damage in the brain. For example, TLR2 upregulation is linked with sterile inflammation, one type being ischemic brain injury. Ziegler et al.^49^ showed that TLR2 acts on glial cells and pro‐inflammatory factors that contribute to the spread of brain injury. Toll‐like receptor 2 has been shown to cause leukocyte infiltration into the injured region via a broken blood‐brain barrier (BBB), as well as the subsequent activation of neuronal death.^50^ Following brain ischemia in mice, TLR2 mRNA levels in resident microglia rises and TLR2 can binds to endogenous ligands. After ischemia injury, High mobility group box 1 (HMGB1), which is regarded as an important damage‐associated molecular pattern (DAMP) in ischemic damage, is localized in the cell nucleus and translocated into the cytosol to activate TLR2.^51^ High‐mobility group box 1 neutralizing antibodies have been shown to decrease infarct volume following ischemic injury.^52^ Another DAMP having a neuroprotective effect is the peroxiredoxin family protein, which is expressed in the damaged region. Neurons of TLR2‐deficient mice are also protected against cell death caused by an ischemia‐like energy deprivation paradigm. In addition, TLR2‐KO animals showed reduced CNS injury after localized CI. These findings imply that the TLR2–CD36 complex is important for inflammatory responses and may operate as a key marker of ischemia at the initiation of death signals. As a result, TLR2 inhibition might be explored in the future as potential therapy against ischemic stroke. Other TLR family members, such as TLR4 are thought to play a key role in the development of BIV in the ischemic brain by binding to endogenous ligands like HMGB1, which trigger immune cell infiltration into the infarct location and its surrounding areas via the BBB. Following cerebral ischemia, TLR4 gene expression is upregulated in neurons, along with an increase in inflammatory cytokines. Knocking down the TLR4 gene in neurons can help them survive in glucose‐depleted environments. This was also seen in TLR4‐deficient animals, which had smaller infarct volumes than WT mice. The TLR4‐deficient animals showed lower levels of cyclooxygenase 2, inducible nitric oxide synthase, and interferon‐γ.^53^

## 4 DISCUSSION

Our meta‐analysis showed that both preC and postC with poly I:C offer substantial neuroprotection against CI. Overall, poly I:C lowers BIV, improves N.S., but does not affect cell death. Following CI, the generation of infarct in the brain is the final pathological step leading to neurological deficits. Injury severity is not directly proportional to the size of the brain infarct.^54^ For example, slight injury in the medial temporal lobe may lead to severe disability such as speech impairment. However, a considerable injury to other parts of the brain may exert a mild functional deficit. Hence, one of the main aims of the CI treatment is functional recovery from neurological damage such as improvement in spasticity or limb impairment.^55^, ^56^ Thereby, N.S. is generally studied together with BIV.^57^ Our N.S. meta‐analysis showed that both preC and postC by poly I:C improve neurological outcomes. Our BIV and N.S. meta‐analysis supports previous findings showing poly I:C exerts neuroprotection by reducing the BIV and N.S.^22^, ^23^, ^24^, ^25^ However, a recent study on role of poly I:C in childhood arteriopathy in mice model showed that poly I:C administration might disrupt the integrity of the BBB and distort the developing neurovascular architecture and vascular networks.^48^ Therefore, the neuroprotective effect of poly I:C lacks generalizability and must be studied in the juvenile model of CI before considering it as an intervention for the different age groups.

Notably, the neuroprotective results of BIV and N.S. displayed a high degree of heterogeneity of I^2^ = 93.52% and 95.63%, respectively. In BIV, the subgroup analysis based on preC versus postC failed to explain the source of heterogeneity. However, subgroup analysis based on species results showed that the heterogeneity significantly decreased in the rat subgroup (I^2^ = 51.35%; considerable heterogeneity to moderate heterogeneity), not in the mice subgroup (I^2^ = 94.80). Thus, it is assumed that BIV outcomes related to mice studies are a significant source of heterogeneity. A probable reason for the moderate heterogeneity in the rat group is that the studies were performed in 2014–2015 while mice studies span over almost a decade. Subsequently, we performed a year‐wise sensitivity analysis for BIV and observed that removing the year 2014 reduces the heterogeneity (I^2^ = 21.35%). This showed that studies performed in the year 2014 are the major source of heterogeneity. Interestingly, our meta‐regression analysis revealed a lack of correlation between dose and BIV, thus eliminating dose‐associated heterogeneity. Similar to BIV the N.S. showed a high level of heterogeneity and species‐wise subgroup analysis showed that high heterogeneity is associated with the mice group while rats showed moderate heterogeneity (I^2^ = 55.45). Furthermore, sensitivity analysis showed that removing reference^34^/the year 2020 reduces the heterogeneity in the N.S. (*p* = 0.00; I^2^=50.26%). The Wang et al.^34^ has high wider CIs in N.S., which is usually associated with the uncertainty of results and might be the reason for this heterogeneity.

Cell death meta‐analysis showed no difference in the level of cell death in poly I:C and vehicle‐treated animals but with a high level of heterogeneity (I^2^ = 97.40%). Sensitivity analysis revealed that cell death results are single‐study driven.^34^ After removal of Wang et al.,^34^ we observed that poly I:C administration significantly reduces cell death (I^2^ = 0.00%; *p* = 0.00). Again, the wider CIs of Wang et al.^34^ in cell death might be the reason for this heterogeneity. These cell death results are consistent with published literature showing that poly I:C treatment significantly reduces brain cell death compared to control,^24^, ^32^ suggesting that poly I:C treatment lowers the cell death after CI.

Our results also suggest that poly I:C significantly upregulates TLR3 levels and prevented ischemia‐induced upregulation of NF‐κB. Likewise, the level of TNF‐α was also downregulated. Also, the results of TLR3, NF‐κB, and TNF‐α showed low heterogeneity, which is an indicator of robust results. These results showed that poly I:C acts through TLR3/NF‐κB/TNF‐α pathway. Our results reject the hypothesis that poly I:C acts independent of TLR3 and supports the previous findings that poly I:C stimulates TLR3,^58^ which downregulates the production of TNF‐α by the NF‐κB signaling pathway.^59^, ^60^

Even though our narrative review indicated that TLR3 can activate the IRF3, our meta‐analysis showed that the level of IRF3 was unaffected by poly I:C administration. This finding is debatable due to the high level of heterogeneity (I^2^ = 94.36%). The heterogeneity of IRF3 declined after removing reference^22^ (I^2^ = 0.00%, *p* = 0.57). Wang et al.^22^ used postC while the remaining two studies^24^, ^32^ used postC, which might be the reason behind this heterogeneity in IRF3. The level of GFAP to understand the effect of poly I:C administration on astrogliosis. Brain ischemia/hypoxia enhanced astrogliosis and excessive astrogliosis leads to impaired neural recovery.^61^ Our results showed poly I:C treatment decreases the level of GFAP expression. This result agrees with the recently published article by Li et al.,^23^ who showed that poly I:C mediated reduction in astrogliosis is neuroprotective.

Our narrative review suggests that TLR2 and TLR4 are known to have a larger role in the pathological progression of ischemic brain injury than other TLRs. As TLR4 suppression can downregulate both MyD88 and TRIF signaling, it can be a powerful neurotherapeutic target. Caso et al.^53^ showed that TLR4‐KO mice have minor infractions and less inflammatory response but no change in IL‐1β and TNF‐α levels after an ischemic insult than wild‐type animals. Subsequently, under ischemic stroke settings, Nalamolu et al.^62^ reported that simultaneous TLR2/TLR4 suppression is more effective than individual suppression, which they conclude is achieved by reducing the production of pro‐inflammatory cytokines TNF, IL‐1, and IL‐6. As a result, TLR2 and TLR4 might be regarded as potential stroke therapeutic targets.

One of the key strengths of this study is combined as well as a separate meta‐analysis of preC and postC of poly I:C. Interestingly, we observed that both preC and postC offer neuroprotection against CI. Moreover, we applied a comprehensive search strategy, had access to the full texts of all identified studies, used the SYRCLE RoB tool to assess the methodological quality of the studies, and relevant extracted data. Furthermore, we performed a detailed subgroup and sensitivity analysis to validate our findings. On the other hand, the postC was performed only in the hyperacute phase of the ischemia, thereby inciting therapeutic uncertainty of poly I:C in later phases of ischemia. Therefore, further research in acute, subacute, and chronic phases of CI is required to establish the therapeutic potential of poly I:C. Another limitation of this work is the high RoB in most of the included studies. Methodological and reporting limitations in reporting/designing are common in animal studies and prevent us from reaching plausible conclusions.^63^, ^64^ Improvements in the preclinical data reporting should be paramount and guidelines regarding the reporting of animal studies should be followed to enhance the quality of research.^32^, ^65^, ^66^ Although the potential source of heterogeneity has been investigated through various subgroup and sensitivity analyses, N.S. showed a moderate level of heterogeneity. Therefore, further research is warranted to establish the neuroprotective role of poly I:C.

## 5 CONCLUSION

Our meta‐analysis showed that preC or postC with poly I:C offers neuroprotection against CI. The findings of this study suggest that poly I:C administration reduces BIV, N.S., and brain cell death. We conclude that poly I:C is a potential therapeutic agent for attenuating neuronal damage and promoting recovery after brain ischemia. We also showed that poly I:C acts via TLR3/NF‐κB/TNF‐α pathway. Our results reveal that TLR3, NF‐κB, and TNF‐α could be utilized as predictive biomarkers for poly I:C treatment against cerebral ischemia injury. Furthermore, our narrative review highlights the importance of using multiple TLRs inhibitors against stroke. Also, it will be interesting to study the combined effect of a TLR activator such as poly I:C with TLR 2/4 inhibitors. TLRs’ molecular structure, genetic differences, and regulation by a variety of reagents can all be used to assist manage stroke prevalence and therapies in the future.

## AUTHOR CONTRIBUTIONS

Y.H. and Z.A.K. performed conceptualization; Z.A.K. performed methodology; Z.A.K., D.M.S., J.C., and G.C.K. performed software; Y.H. and Z.A.K. validated the document; Z.A.K., D.M.S., J.C., and G.C.K. performed formal analysis; Z.A.K. and D.M.S. investigated the data; Y.H. performed resources; Z.A.K., D.M.S., and J.C. performed data curation; Y.H. and Z.A.K. wrote original draft preparation; Y.H. and Z.A.K. reviewed and edited; Z.A.K. and D.M.S. performed visualization; Y.H. supervised the data; J.C. involved in project administration; Y.H. contributed to funding acquisition. All authors read and approved the final manuscript.

## CONFLICT OF INTEREST

The authors declare no conflict of interest.

## Supporting information

Supplementary Material

## Data Availability

The datasets used and/or analyzed during the current study are available from the corresponding author on reasonable request.
