# Supplementary material for: Pre‐ and post‐conditioning with poly I:C exerts neuroprotective effect against cerebral ischemia injury in animal models: A systematic review and meta‐analysis
Source: CNS Neurosci Ther. 2022 May 5;28(8):1168–82. doi: 10.1111/cns.13851 (PMC9253751; doi:10.1111/cns.13851)
Supplement: Supplementary file 1 — Supplementary Material [file CNS-28-1168-s001.docx]

# SUPPLEMENTAL DATA

**Contents:**

**Supplementary Tables**

**Supplementary Table S1:** PubMed search strategy (08 March 2021).

**Supplementary Table S2:** Embase search strategy (08 March 2021).

**Supplementary Table S3:** Explanations for the full-text article exclusions.

**Supplementary Table S4:** Leave-one-out sensitivity analyses on brain infarct volume.

**Supplementary Table S5:** Year-wise sensitivity analyses on brain infarct volume.

**Supplementary Table S6:** Leave-one-out sensitivity analyses on neurological score.

**Supplementary Table S7:** Year-wise sensitivity analyses on the neurological score.

**Supplementary Table S8:** Leave-one-out sensitivity analyses on cell death.

**Supplementary Table S9:** Leave-one-out sensitivity analyses on NF-κB.

**Supplementary Table S10:** Leave-one-out sensitivity analyses on IRF3.

**Supplementary Figures**

**Supplementary Figure S1:** Bubble plot with a fitted meta-regression line of the relationship between the poly I:C doses with BIV.

**Supplementary Figure S2:** Subgroup analysis of BIV levels between poly I:C and vehicle-treated groups according to species.

**Supplementary Figure S3:** Subgroup analysis of N.S. levels between poly I:C and vehicle-treated groups according to species.

**Supplementary Figure S4:** Subgroup analysis of BIV levels between poly I:C and vehicle-treated groups according to time of intervention.

**Supplementary Figure S5:** Subgroup analysis of N.S. levels between poly I:C and vehicle-treated groups according to time of intervention.

**Supplementary Figure S6:** Forest plot comparing change in brain cell death between poly I:C and vehicle-treated groups.

**Supplementary Figure S7:** a) Funnel plot showing the variation of effect size distribution for BIV outcome with imputed studies adjusting for publication bias. b) funnel plot showing the symmetrical distribution of studies indicating the absence of publication bias after removing the outliers.

**Supplementary Figure S8:** Forest plot comparing changes in BIV in the brain between poly I:C and vehicle treated group after cerebral ischemia without outlier studies.

**Supplementary Figure S9:** Funnel plot of studies reporting the Neurological deficit score after cerebral ischemia a) without and b) with imputed studies adjusting for publication bias.

**Supplementary Tables:**

**Supplementary Table S1:** PubMed search strategy (08 March 2021).

| **Search** | **Query** | **Items found** |
| --- | --- | --- |
| #1 | ("poly i c"[MeSH Terms] OR ("poly"[All Fields] AND "i c"[All Fields]) OR "poly i c"[All Fields] OR ("polyinosinic"[All Fields] AND "polycytidylic"[All Fields] AND "acid"[All Fields]) OR "polyinosinic polycytidylic acid"[All Fields] OR polyICLC OR poly I:C12U) | 8,478 |
| #2 | (("hypoxia"[MeSH Terms]) OR ("hypoxia"[All Fields]) OR ("hypoxia"[Title/Abstract])) OR ("hypoxia"[Text Word]) OR hypox* ("oxygen deficiency"[Title/Abstract]) OR ("oxygen deficiency"[Text Word]) OR ("oxygen deficiencies"[Text Word]) OR ("oxygen deficiency"[Title]) OR (("ischemia"[MeSH Terms]) OR ("ischemia"[Title/Abstract])) OR (ischemia[Text Word]) OR 'Ischemia[Text Word] OR Reperfusion'[Text Word] OR 'Ischemia[Text Word] OR Reperfusion'[Text Word] OR Ischem* (("oxygen inhalation therapy"[MeSH Terms]) OR ("oxygen inhalation therapy"[Title/Abstract])) OR ("oxygen inhalation therapy"[Text Word]) OR ("hypoxemia"[Title/Abstract]) OR ("hypoxemia"[Text Word]) OR ("anoxia"[Text Word]) OR ("anoxia"[Title/Abstract]) OR ("anoxemia"[Text Word]) OR ("anoxemia"[Title/Abstract]) OR (("hypoxia inducible factor"[Title/Abstract]) OR ("hypoxia inducible factor 1"[MeSH Terms])) OR ("hypoxia inducible factor"[Text Word]) OR ("hif"[Title/Abstract]) OR ("hif"[Text Word]) OR "hif 2"[Title/Abstract] OR "hif 3"[Title/Abstract] OR (("ischemia"[MeSH Terms]) OR ("ischemia"[Text Word])) OR ("ischemia"[Title/Abstract]) OR ("no reflow phenomenon"[Text Word]) OR ("no reflow phenomenon"[Title/Abstract]) OR (("infarction"[MeSH Terms]) OR ("infarction"[Text Word])) OR (infarction[Title/Abstract]) | 621,545 |
| #3 | #1 AND #2 | 49 |

**Supplementary Table S2:** Embase search strategy (08 March 2021).

| **Search** | **Query** | **Items found** |
| --- | --- | --- |
| #1 | ('polyinosinic polycytidylic acid'/exp OR 'polyinosinic polycytidylic acid' OR '24939 03 5') | 9281 |
| #2 | ('hypoxia'/exp OR hypox*:ab,ti,kw OR hif:ab,ti,kw OR 'oxygen'/exp OR deficiency, OR oxygen:ab,ti,kw OR hypoxia:ab,ti,kw OR hypoxia OR warning:ab,ti,kw OR diffusion OR anoxia:ab,ti,kw OR hypoxic) AND drive:ab,ti,kw OR hypoxic OR blood OR flow OR disorder:ab,ti,kw OR circulation OR failure:ab,ti,kw OR ischemic OR disease:ab,ti,kw OR 'ischem*':ab,ti,kw OR circulatory OR disturbance:ab,ti,kw OR ischaemia:ab,ti,kw OR 'ischaemic disease':ab,ti,kw OR 'ischaemic syndrome':ab,ti,kw OR 'tissue ischaemia':ab,ti,kw OR 'warm ischaemia':ab,ti,kw OR 'warm ischaemia time':ab,ti,kw OR 'warm ischemia':ab,ti,kw OR 'warm ischemia time stress':ab,ti,kw OR 'oxygen deficiency':ab,ti,kw OR 'hypoxemia':ab,ti,kw OR 'reperfusion':ab,ti,kw OR 'infarction':ab,ti,kw OR hif:ab,ti,kw) | 1,082, 097 |
| #3 | #1 AND #2 | 115 |

**Supplementary Table S3.** Explanations for the full-text article exclusions.

| SL/NO | Title | Reasons |
| --- | --- | --- |
| 1 | TLR3 contributes to persistent autophagy and heart failure in mice after myocardial infarction | Unrelated population |
| 2 | Modulation of innate immune responses in Atlantic salmon by chronic hypoxia-induced stress | Unrelated population |
| 3 | TLR-3 mediated preconditioning protects the immature brain from ischemic injury | Meeting abstract |
| 4 | TLR3 ligand attenuates cerebral ischemia/reperfusion injury | Meeting abstract |
| 5 | Implicating Receptor Activator of NF-κB (RANK)/RANK Ligand Signalling in Microglial Responses to Toll-Like Receptor Stimuli | Unrelated study design |
| 6 | Antiviral activity of polynucleotides: copolymers of inosinic acid and N2 dimethylguanylic or 2 methylthioinosinic acid | Unrelated outcome |
| 7 | HIF-1alpha-dependent gene expression program during the nucleic acid-triggered antiviral innate immune responses | Unrelated outcome |
|  |  |  |

**Supplementary Table S4: Leave-one-out sensitivity analyses on Brain infarct volume**

|  | **Pooled SMD [95%CI]** | **Between-study heterogeneity** |
| --- | --- | --- |
| **Overall** | -2.31 [-3.63, -0.99] | *p* =0.00; I^2^=93.52% |
| **Omitted study** | | |
| Packard 2011 | -2.38 [-3.83, -0.93] | *p* =0.00; I^2^=94.02% |
| Pan 2012 | -2.25 [-3.68, -0.82] | *p* =0.00; I^2^=94.20% |
| Shi 2013 a | -2.40 [-3.84, -0.95] | *p* =0.00; I^2^=93.94% |
| Shi 2013 b | -2.55 [-3.89, -1.21] | *p* =0.00; I^2^=92.74% |
| Stridh 2013 | -2.62 [-3.80, -1.45] | *p* =0.00; I^2^=89.84% |
| Wang 2014 a | -2.14 [-3.52, -0.76] | *p* =0.00; I^2^=93.82% |
| Wang 2014 b | -2.31 [-3.75, -0.87] | *p* =0.014; I^2^=74.63% |
| Zhang 2014 a | -2.41 [-3.85, -0.97] | *p* =0.00; I^2^=94.00% |
| Zhang 2014 b | -2.35 [-3.80, -0.90] | *p* =0.00; I^2^=94.21% |
| Gesuete 2015 | -2.27 [-3.71, -0.83] | *p* =0.00; I^2^=94.22% |
| Jeong 2015 | -2.09 [-3.43, -0.76] | *p* =0.00; I^2^=93.56% |
| Li 2015 | -2.42 [-3.86, -0.99] | *p* =0.00; I^2^=94.02% |
| Wang 2020 | -1.85 [-2.92, -0.78] | *p* =0.00; I^2^=90.17% |

**Supplementary Table S5: Year-wise sensitivity analyses on Brain infarct volume.**

|  | **Pooled SMD [95%CI]** | **Between-study heterogeneity** |
| --- | --- | --- |
| **Overall** | -2.31 [-3.63, -0.99] | *p* =0.00; I^2^=93.52% |
| **Omitted study** | | |
| 2011 | -2.38 [-3.83, -0.93] | *p* =0.00; I^2^=94.02% |
| 2012 | -2.25 [-3.68, -0.82] | *p* =0.00; I^2^=94.20% |
| 2013 | -3.06 [-4.25, -1.87] | *p* =0.00; I^2^=85.76% |
| 2014 | -2.29 [-4.22, -0.36] | *p* =0.00; I^2^=24.47% |
| 2015 | -2.15 [-3.78, -0.51] | *p* =0.00; I^2^=94.98% |
| 2020 | -1.85 [-2.92, -0.78] | *p* =0.00; I^2^=90.17% |

**Supplementary Table S6: Leave-one-out sensitivity analyses on neurological score**

|  | **Pooled SMD [95%CI]** | **Between-study heterogeneity** |
| --- | --- | --- |
| **Overall** | -2.50 [-4.76, -0.24] | *p* = 0.00; I^2^=95.63% |
| **Omitted study** | | |
| Packard 2011 | -2.53 [-5.40, 0.35] | *p* = 0.00; I^2^=96.91% |
| Pan 2012 | -2.81[-5.63, 0.02] | *p* = 0.00; I^2^=96.35% |
| Wang 2014 a | -2.78 [-5.62, -0.06] | *p* = 0.00; I^2^=96.52% |
| Wang 2014 b | -2.78 [-2.78, 0.06] | *p* = 0.00; I^2^=96.42% |
| Li 2015 | -2.98 [-5.63, -0.32] | *p* = 0.00; I^2^=95.78% |
| Wang 2020 | -1.29 [-1.75, -0.83] | *p* = 0.00; I^2^=50.82% |

**Supplementary Table S7: Year-wise sensitivity analyses on the neurological score.**

|  | **Pooled SMD [95%CI]** | **Between-study heterogeneity** |
| --- | --- | --- |
| **Overall** | -2.50 [-4.76, -0.24] | *p* = 0.00; I^2^=95.63% |
| **Omitted study** | | |
| 2011 | -2.53 [-5.40, 0.35] | *p* = 0.00; I^2^=96.91% |
| 2012 | -2.81 [-5.63, 0.02] | *p* = 0.00; I^2^=96.35% |
| 2014 | -3.20 [-6.90, 0.50] | *p* = 0.00; I^2^=97.09% |
| 2015 | -2.98 [-5.63, -0.32] | *p* = 0.00; I^2^=95.78% |
| 2020 | -1.35 [-2.01, -0.69] | *p* = 0.00; I^2^=50.26% |

**Supplementary Table S8: Leave-one-out sensitivity analyses on Cell death**

|  | **Pooled SMD [95%CI]** | **Between-study heterogeneity** |
| --- | --- | --- |
| **Overall** | -4.53 [-10.59, 1.54] | *p* = 0.00; I^2^=97.40% |
| **Omitted study** | | |
| Wang 2014 | -6.33 [-15.53, 2.88] | *p* = 0.00; I^2^=95.18% |
| Zhang 2014 | -6.07 [-15.76, 3.61] | *p* =0.00; I^2^= 95.95% |
| Wang 2020 | -1.46 [-2.31, -0.61] | *p* =0.60; I^2^= 0.00% |

**Supplementary Table S9:** Leave-one-out sensitivity analyses on NF-κB.

|  | **Pooled SMD [95%CI]** | **Between-study heterogeneity** |
| --- | --- | --- |
| **Overall** | -1.78 [-2.67, -0.88] | *p* = 0.54; I^2^=0.00% |
| **Omitted study** | | |
| Shi 2013 | -1.54 [-2.54, -0.53] | *p* = 0.68; I^2^=0.00% |
| Wang 2014 | -1.81 [-3.05, -0.58] | *p* = 0.27; I^2^=18.75% |
| Zhang 2014 | -2.18 [-3.44, -0.91] | *p* = 0.49; I^2^=0.00% |

**Supplementary Table S10:** Leave-one-out sensitivity analyses on IRF3.

|  | **Pooled SMD [95%CI]** | **Between-study heterogeneity** |
| --- | --- | --- |
| **Overall** | 3.62 [-1.24, 8.47] | *p* = 0.01; I^2^=94.36% |
| **Omitted study** | | |
| Shi 2013 | 5.49 [-2.80, 13.77] | *p* = 0.01; I^2^=86.67% |
| Wang 2014 | 1.47 [0.48, 2.46] | *p* = 0.57; I^2^=0.00% |
| Zhang 2014 | 5.24 [-3.63, 14.10] | *p* = 0.00; I^2^=88.22% |

**Supplementary Figures:**

**Supplementary Figure S1:** Bubble plot with a fitted meta-regression line of the relationship between the poly I:C doses with BIV. Circles are sized according to the precision of each estimate (the inverse of its within-study variance). BIV, brain infarct volume; poly I:C, Polyinosinic:polycytidylic acid.


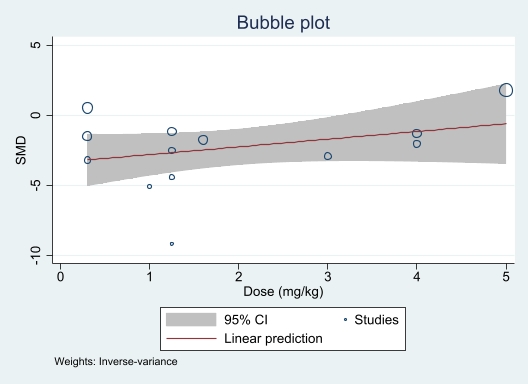


**Supplementary Figure S2:** Subgroup analysis of BIV levels between poly I:C and vehicle-treated groups according to species. The test for subgroup differences showed no statistically significant subgroup effect, indicating that species did not modify the BIV as compared with vehicle-treated groups following cerebral ischemia. The prism represents the overall statistical results of the experimental data, squares represent the weight of each study, and horizontal lines represent the 95% CIs for each study. BIV, brain infarct volume; poly I:C, Polyinosinic:polycytidylic acid.; CIs, Confidence intervals; SD, Standard deviation; IV, Independent variable.


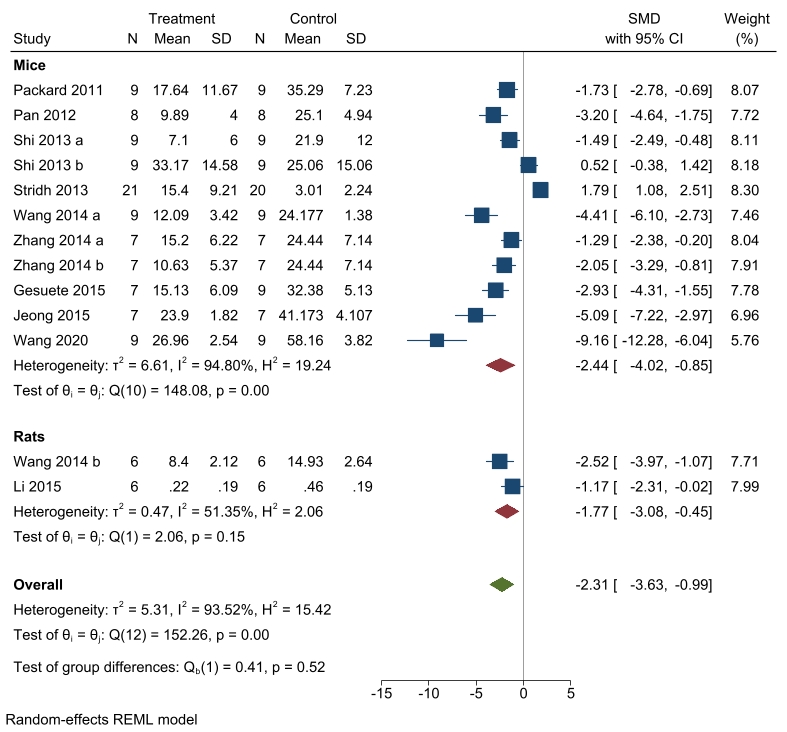


**Supplementary Figure S3:** Subgroup analysis of N.S. levels between poly I:C and vehicle-treated groups according to species. The test for subgroup differences showed no statistically significant subgroup effect, indicating that species did not modify the N.S. as compared with vehicle-treated groups following cerebral ischemia. The prism represents the overall statistical results of the experimental data, squares represent the weight of each study, and horizontal lines represent the 95% CIs for each study. N.S., Neurological deficit score; poly I:C, Polyinosinic:polycytidylic acid.; CIs, Confidence intervals; SD, Standard deviation; IV, Independent variable.


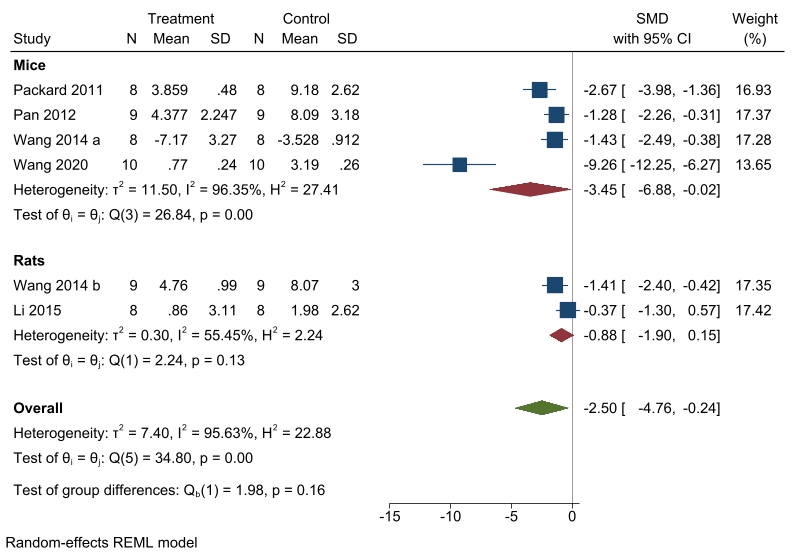


**Supplementary Figure S4:** Subgroup analysis of BIV levels between poly I:C and vehicle-treated groups according to time of intervention. The test for subgroup differences showed no statistically significant subgroup effect, indicating that species did not modify the BIV as compared with vehicle-treated groups following cerebral ischemia. The prism represents the overall statistical results of the experimental data, squares represent the weight of each study, and horizontal lines represent the 95% CIs for each study. BIV, brain infarct volume; poly I:C, Polyinosinic:polycytidylic acid.; CIs, Confidence intervals; SD, Standard deviation; IV, Independent variable.


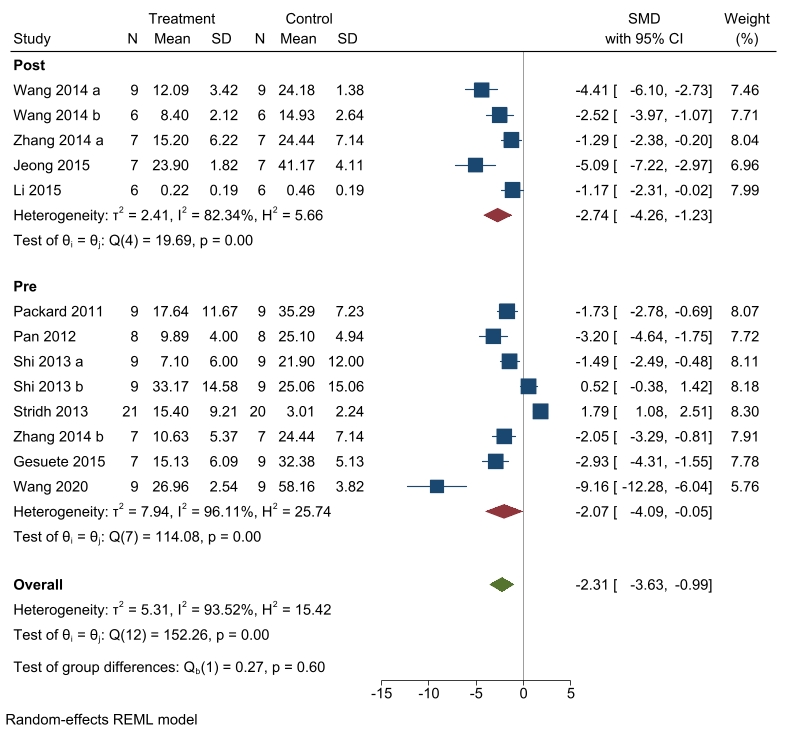


**Supplementary Figure S5:** Subgroup analysis of N.S. levels between poly I:C and vehicle-treated groups according to time of intervention. The test for subgroup differences showed no statistically significant subgroup effect, indicating that species did not modify the N.S. as compared with vehicle-treated groups following cerebral ischemia. The prism represents the overall statistical results of the experimental data, squares represent the weight of each study, and horizontal lines represent the 95% CIs for each study. N.S., Neurological deficit score; poly I:C, Polyinosinic:polycytidylic acid; CIs, Confidence intervals; SD, Standard deviation; IV, Independent variable.


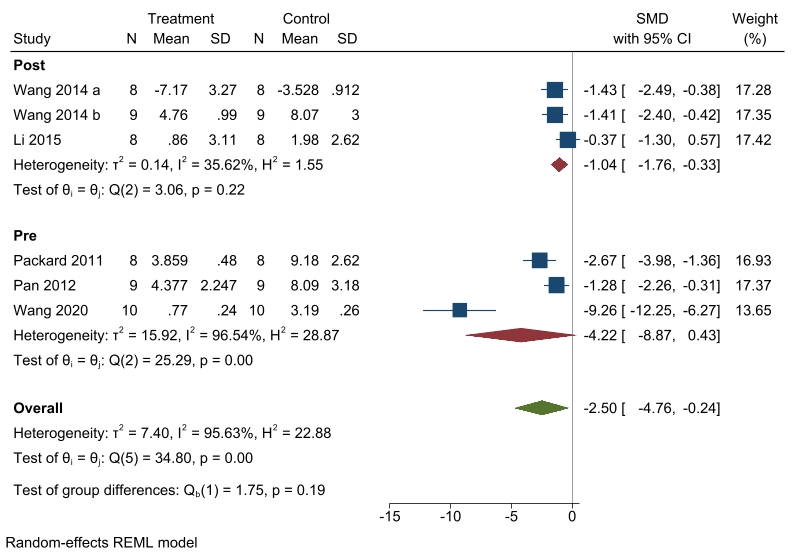


**Supplementary Figure S6:** Forest plot comparing change in brain cell death between poly I:C and vehicle-treated groups without Wang *et al.* ^42^. Statistically significant change was observed in the brain cell death when the study Wang et al. 42 was removed from the meta-analysis. Also, heterogeneity was reduced to only 0.00%. The prism represents the overall statistical results of the experimental data, squares represent the weight of each study, and horizontal lines represent the 95% CIs for each study. poly I:C, Polyinosinic:polycytidylic acid; CIs, Confidence intervals; CHI, SD, Standard deviation; IV, Independent variable.


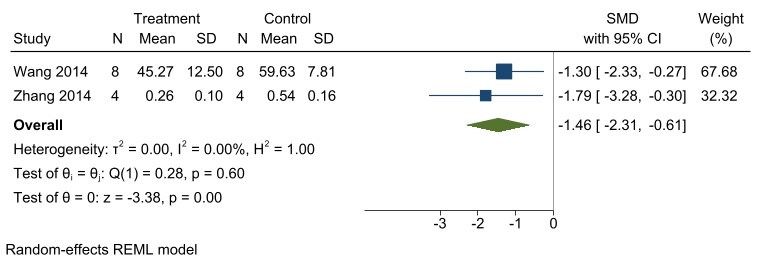


**Supplementary Figure S7:** a) Funnel plot showing the variation of effect size distribution for BIV outcome with imputed studies adjusting for publication bias. b) funnel plot showing the symmetrical distribution of studies indicating the absence of publication bias after removing the outliers.

| 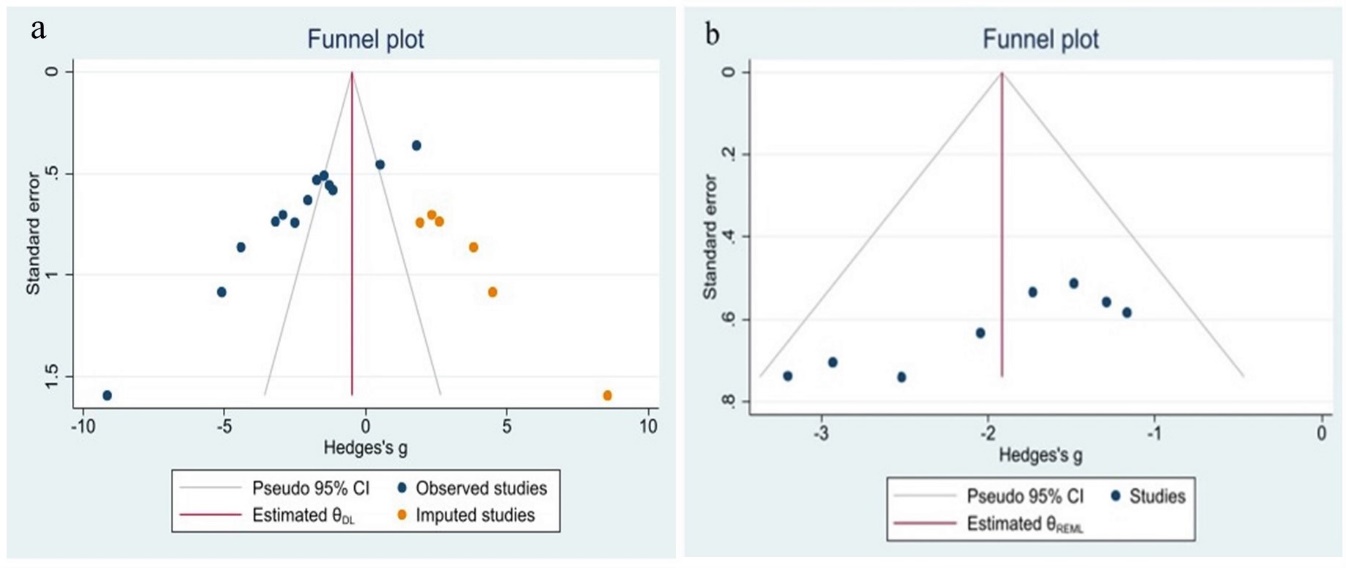 |
| --- |

**Supplementary Figure S8:** Forest plot comparing changes in BIV in the brain between poly I:C and vehicle-treated group after cerebral ischemia without outlier studies. BIV was significantly reduced in poly I:C group compared with the vehicle group. The heterogeneity was reduced to 21.35%. The prism represents the overall statistical results of the experimental data, squares represent the weight of each study, and horizontal lines represent the 95% CIs for each study. BIV, brain infarct volume; poly I:C, Polyinosinic:polycytidylic acid.; CIs, Confidence interval; SD, Standard deviation; IV, Independent variable.


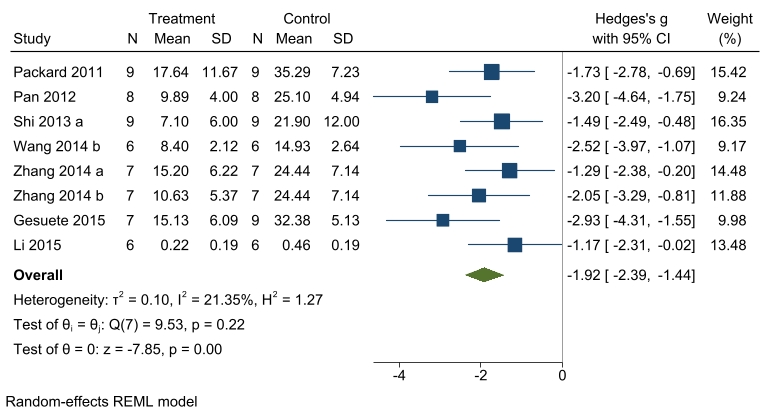


**Supplementary Figure S9:** Funnel plot of studies reporting the Neurological deficit score after cerebral ischemia a) without and b) with imputed studies adjusting for publication bias.

| 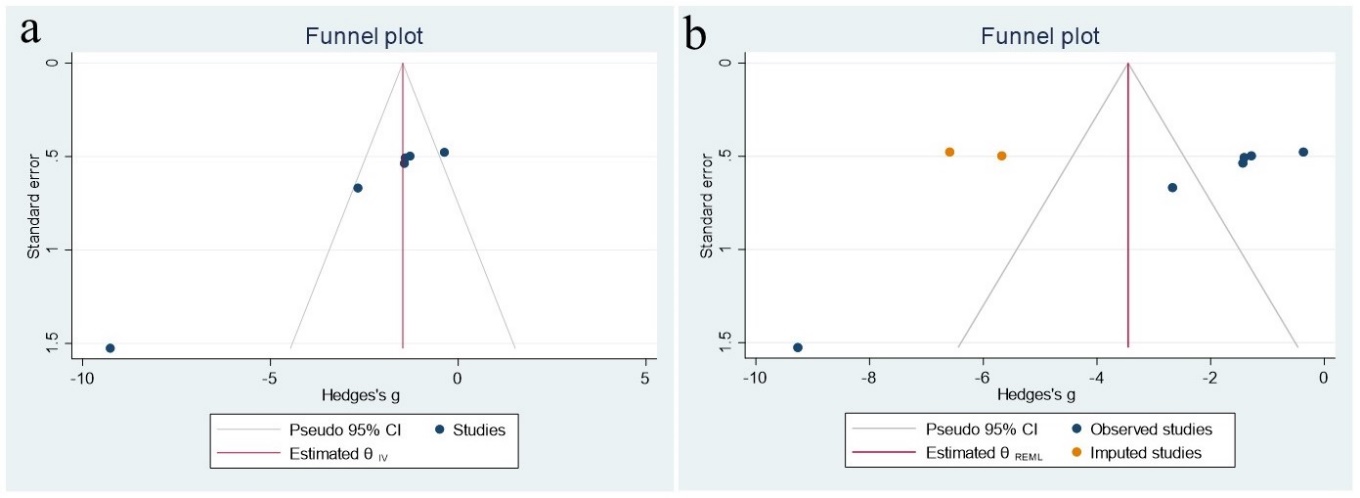 |
| --- |
